# Preparation of Antioxidant Enzymatic Hydrolysates from Honeybee-Collected Pollen Using Plant Enzymes

**DOI:** 10.4061/2010/415949

**Published:** 2011-01-09

**Authors:** Margarita D. Marinova, Bozhidar P. Tchorbanov

**Affiliations:** Institute of Organic Chemistry with Centre of Phytochemistry, Bulgarian Academy of Sciences, Acad. G. Bonchev Street, Building 9, BG-1113 Sofia, Bulgaria

## Abstract

Enzymatic hydrolysates of honeybee-collected pollen were prepared using food-grade proteinase and aminopeptidases entirely of plant origin. Bromelain from pineapple stem was applied (8 mAU/g substrate) in the first hydrolysis stage. Aminopeptidase (0.05 U/g substrate) and proline iminopeptidase (0.03 U/g substrate) from cabbage leaves (*Brassica oleracea * var. *capitata*), and aminopeptidase (0.2 U/g substrate) from chick-pea cotyledons (*Cicer arietinum* L.) were involved in the additional hydrolysis of the peptide mixtures. The degree of hydrolysis (DH), total phenolic contents, and protein contents of these hydrolysates were as follows: DH (about 20–28%), total phenolics (15.3–27.2 *μ*g/mg sample powder), and proteins (162.7–242.8 *μ*g/mg sample powder), respectively. The hydrolysates possessed high antiradical scavenging activity determined with DPPH (42–46% inhibition). The prepared hydrolysates of bee-collected flower pollen may be regarded as effective natural and functional dietary food supplements due to their remarkable content of polyphenol substances and significant radical-scavenging capacity with special regard to their nutritional-physiological implications.

## 1. Introduction

Natural products and preparations for food and nutritional supplementation or dietary purposes have gained increased attention in recent years. Among them, honeybee-derived apicultural products, such as pollen, have been applied for centuries in alternative medicine as well as in food diets and supplementary nutrition due to their nutritional and physiological properties. Each pollen has its own specificity, mainly linked to the floral species or cultivars. Bee-collected pollens contain nutritionally essential substances including carbohydrates, proteins, amino acids, lipids, vitamins, mineral substances, and trace elements, but also significant amounts of polyphenol substances mainly flavonoids which, furthermore, are regarded as principal indicating ingredient substances of pollen and can be used for setting up quality standards in relation to their nutritional-physiological properties and for quality control of commercially distributed pollen preparations [1–4]. It is well known that polyphenols are responsible for the antioxidative and radical scavenging activity of plant food [5, 6]. An antioxidant defense system protects cells from the injurious effects of free radicals. Furthermore, the biological, biochemical, physiological, pharmaceutical, and medicinal properties of polyphenol compounds have been extensively studied and have been reviewed by Rice-Evans et al. [7] in regard to their free-radical scavenging activity and multiple biological activities including vasodilatory, anticarcinogenic, anti-inflammatory, antibacterial, immune-stimulating, antiallergic, antiviral, and estrogenic effects, as well as being inhibitors of specific enzymes. 

On the basis of these reports, we have prepared water-soluble fractions from honeybee-collected pollen and investigated their functional properties. As a result, high free-radical scavenging activities against the DPPH free radical were exhibited. These results are comparable to the results reported for the antioxidant activities in red grapes (*Vitis vinifera*, L.) extracts [8, 9]. Moreover, we showed that enzymatic hydrolysates from honeybee-collected pollens possessed even higher antioxidative properties. In the present study, our aim was to prepare enzymatic hydrolysates from honeybee-collected pollens using plant proteinase and aminopeptidases and, then, to investigate the antioxidant activities in these peptide samples.

## 2. Materials and Methods

### 2.1. Materials

The pollen loads were collected in 2009 from the Ajtos area by honeybee colonies (*Apis mellifera*) settled in hives with bottom-fitted pollen traps. The aminopeptidase and proline iminopeptidase from cabbage leaves (*Brassica oleracea *var.* capitata*) and the aminopeptidase from chick-pea cotyledons (*Cicer arietinum *L.) were purified as described by Marinova et al. [10, 11]. Bromelain from pineapple stems (EC 3.4.22.4, 2 mAnsonU/mg), L-amino acid *p*-nitroanilides, Folin-Ciocalteu, and 1,1-diphenyl-2-picrylhydrazyl (DPPH), were purchased from Sigma-Aldrich Co. (St. Louis, USA).

### 2.2. Methods

#### 2.2.1. Preparation of Enzymatic Hydrolysates from Honeybee-Collected Pollen

Honeybee-collected pollens (28% protein) were added, suspended in 5 volumes of distilled water, and homogenized (Ultra-Turax, IKA-Werke, Germany), and pH of the suspension was adjusted at 7.0 using NH_4_OH. The digestion was started by addition of 8 mAU/g bromelain at 37°C. After 4 hours, hydrolysis was stopped by boiling in a microwave for 2 minutes. The additional hydrolysis was carried out by adding aminopeptidase (0.05 units/g substrate) and proline iminopeptidase (0.03 units/g substrate) from cabbage leaves as well as aminopeptidase from chick-pea cotyledons (0.2 units/g substrate) and incubating for two hours at 37°C and pH 7.5 with constant stirring. Hydrolysis was stopped by boiling in a microwave for 2 minutes. The obtained hydrolysates were centrifuged at 6000 × g for 30 min at 5°C (MLW K24 D, Germany) to remove the residue. The supernatant fractions were collected and freeze-dried.

#### 2.2.2. Assays of Enzymes' Activities, Total Nitrogen, Total Protein, and Total Phenolic Compounds

Aminopeptidases' activities were determined using L-leucine-*p*-nitroanilides as substrate [12]. After incubation for 10 min at 30°C in 0.05 M sodium phosphate buffer (pH 7.2–7.5), the liberated *p*-nitroaniline was measured at 410 nm on a spectrophotometer (UV-VIS Spectrophotometer, Shimadzu 1240). The iminopeptidase activity was assayed spectrophotometrically at 410 nm against L-proline-*p*-nitroanilide (Pro-*p*-NA) in 0.1 M Tris/HCl buffer (pH 8.0) for 20 min at 30°C [13]. One unit of enzyme activity was defined as the amount of enzyme releasing 1 *μ*mol of *p*-nitroaniline per minute.

The total protein content of the honeybee-collected pollen was determined by the method of Kjeldahl using the equation: *N* × 6.25, where *N* is the total Kjeldahl nitrogen multiplied by a factor to arrive at protein content [14]. The protein concentration of the samples after hydrolysis was measured according to the method of Lowry et al. [15], using bovine serum albumin as standard. The total phenolic content was determined by the Folin-Ciocalteu colorimetric method using catechin as standard, and the absorbance was measured at 760 nm [16].

#### 2.2.3. Radical Scavenging Activity

The antiradical power of bee-collected pollen and pollen hydrolysates was evaluated in terms of the hydrogen-donating or radical-scavenging ability by the DPPH method [17], which is related to the inhibition in the initiation step of free radical processes. DPPH (2,2-diphenyl-1- picrylhydrazyl) is a stable free radical that accepts an electron or hydrogen radical to become a stable diamagnetic molecule and, accordingly, is reduced in presence of an antioxidant (AH):

(1) $${\text{DPPH}}^{•}+\text{AH}\to \text{DPPH}-\text{H}\;+\;{\text{A}}^{•}\cdot$$

For the evaluation of the antioxidant activity of specific compounds or extracts, they are allowed to react with the stable DPPH radical in a methanol solution. In its radical form, DPPH has a characteristic absorbance at 515 nm, which disappears upon reduction by H gained from an antioxidant compound. 

For the test, appropriate methanol stock solutions of the pollen preparations (500 mg/L) and DPPH (6 × 10^−5^ mol/L) were prepared. Immediately after adding 0.3 mL of the pollen extract solution to 2.7 mL of the DPPH solution, the reduction of the DPPH-radical was measured by monitoring continuously the decrease of absorption at 515 nm in the dark until stable absorption values were obtained (30 min). The antiradical activity was determined in terms of PI values (% inhibition) which was calculated by the ratio of the decrease of absorption of the DPPH-pollen extract test solution after a 30-minute reaction time (stable phase) to the absorption value of the reference sample where an equivalent volume of methanol was added, as defined according to the formula:

(2) $$\text{PI}\;\left(\text{\%}\;\text{inhibition}\right)=\left[A_{0}-\frac{A_{\text{t}}}{A_{0}}\right]\times 100,$$

where *A*_0_ is the absorbency of the DPPH-methanol solution (reference) and *A*_*t*_ is the absorbency of the DPPH-pollen extract solution after 30 min of reaction time.

#### 2.2.4. SDS-Polyacrylamide Gel Electrophoresis

Sodium dodecyl sulfate polyacrylamide gel electrophoresis (SDS-PAGE) was performed in 15% polyacrylamide gel using Tris-glycine buffer, pH 8.3, according to Laemmli [18]. Rabbit muscle myosin (205 kDa), *β*-galactosidase (116 kDa), rabbit muscle phosphorylase b (97 kDa), bovine serum albumin (66 kDa), lactate dehydrogenase (36.5 kDa), carbonic anhydrase (29 kDa), trypsin inhibitor (20 kDa), lysozyme (14 kDa), aprotinin (6.1 kDa), insulin a (3.4 kDa), and insulin b (2.4 kD) were used as molecular weight marker proteins. The gel was visualized by silver staining [19].

#### 2.2.5. Determination of the Degree of Hydrolysis (DH) and Amino Acid Composition

The degree of hydrolysis was determined using 2,4,6-trinitrobenzenesulfonic acid [20]. A sample solution (0.25 mL) is mixed with 2.0 mL of 0.2 M sodium phosphate buffer (pH 8.2) and 2.0 mL of 0.1% trinitrobenzenesulfonic acid, followed by incubation in the dark for 60 min at 50°C. The reaction is quenched by adding 4.0 mL of 0.1 N HCl, and the absorbance is read at 340 nm. A 1.5 mM L-leucine solution is used as the standard. Transformation of the measured leucine amino equivalents to degree of hydrolysis is carried out by means of a standard curve for each particular protein substrate.

The amino acid composition was determined after 50 min of hydrolysis at 165°C with 6 N HCl, and the analysis was performed on HPLC Nova-Pak C18 (3.9 × 150 mm, 4 *μ*m, Waters). The mobile phase consisted of eluent A (prepared from Waters AccQ·Tag Eluent A concentrate, by adding 200 mL of concentrate to 2 L of Milli-Q water and mixing), eluent B (acetonitrile, HPLC grade), and eluent C (Milli-Q water). The following conditions were used: linear gradient of 100–0% eluent A, 0–60% eluent B, and 0–40% eluent C in 30 min and then isocratic 100% of eluent A for 20 min with a flow rate of 1 mL/min.

## 3. Results and Discussion

### 3.1. The Total Phenolic Contents and Protein Contents of Enzymatic Hydrolysates from Bee Pollen

**Figure 1:**
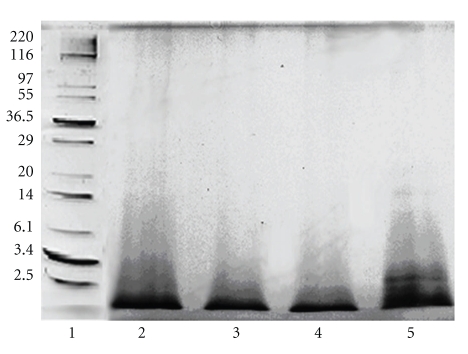
SDS-polyacrylamide gel electrophoresis of molecular weight markers and enzymatic hydrolysates from honeybee-collected pollen. (1) Molecular weight markers; (2) aminopeptidases hydrolysate (cabbage and chick-pea); (3) aminopeptidase and proline iminopeptidase hydrolysate (cabbage); (4) aminopeptidase hydrolysate (chick-pea); (5) bromelain hydrolysate.

The enzymatic hydrolysates from bee pollen were digested and prepared using plant proteinase bromelain, and aminopeptidases from cabbage leaves and chick-pea cotyledons. SDS-PAGE analysis indicated that the pollen was perfectly digested by these enzymes (Figure 1). The degrees of hydrolysis of the bee-pollen hydrolysates were as follows: about 20% for the bromelain hydrolysate (BH), 26% for the cabbage aminopeptidase and proline iminopeptidase hydrolysate (APH_1_), 24% for the chick-pea aminopeptidase hydrolysate (APH_2_), and 28% for the hydrolysate obtained by the combination of aminopeptidases from cabbage and chick-pea (APH_3_). Total phenolic contents of these hydrolysates were as follows: 21.5 *μ*g/mg sample (BH), 25.6 *μ*g/mg sample (APH_1_), 24.1 *μ*g/mg sample (APH_2_), and 27.2 *μ*g/mg sample (APH_3_), respectively (Table 1). On the other hand, the protein contents of these hydrolysates were as follows: 227.1 *μ*g/mg sample (BH), 238.8 *μ*g/mg sample (APH_1_), 230.5 *μ*g/mg sample (APH_2_), and 242.8 *μ*g/mg sample (APH_3_), respectively (Table 1). It suggests that the protein contents correlated closely with the contents of total phenolic components.

### 3.2. DPPH Radical Scavenging Ability of Enzymatic Hydrolysates from Pollen

Although native bee-collected pollen water extract shows considerable antiradical activity (PI = 28% inhibition), the reduction of the DPPH radical was significantly increased by applying the obtained pollen hydrolysates (PI = 42–46% inhibition) as is shown in Table 1, which indicate an elevated free-radical scavenging efficiency of the pollen hydrolysates. The highest degree of radical scavenging capacity was assessed in the APH_3_ (PI = 46% inhibition) which, correspondingly, also has the highest concentration of polyphenol substances (27.2 *μ*g/mg sample powder). For this reason, it can be assumed that there is a general correlation between the content of total polyphenolics and the free-radical scavenging capacity of the pollen preparations. 

Although, in comparison, tests with equivalent amount of the synthetic antioxidant gallic acid shows higher PI values of approximately 90%, it must be taken into consideration that pollen extract represents a concentrated nature-derived mixture of different active polyphenol compounds. 

The results of the amino acids' composition for the bee-pollen extract and the pollen hydrolysate obtained by combination of aminopeptidases from cabbage and chick-pea are shown in Table 2. Relatively high content of hydrophobic amino acids Pro, Phe, and Gly is characteristic for the bee-pollen extract and the APH_3_. The amounts of these amino acids in bee-pollen hydrolysate after a 6-hour hydrolysis are approximately 22, 5.5 and 10.5%, respectively of the total content.

The honeybee products are considered to be abundant sources of antioxidants. In honey, royal jelly, propolis, and bee pollens high antioxidant activity was found [21]. In bee-collected pollen water extracts high radical-scavenging activity, activity against superoxide anion, and hydroxyl radical-scavenging activity were reported [22–25]. 

Antioxidative ability of pollen seems to be due to phenolic compounds. In the present investigations, a very high antioxidant activity, expressed as radical-scavenging activity corresponded to high levels of total phenols, was found in water-soluble extract and in plant proteinase and aminopeptidases hydrolysates.

## 4. Conclusion

The use of honeybee-collected pollens as an alternative medicine is increasing due to their biologically active properties that make them attractive as a source of essential amino acids, vitamins, minerals, and antioxidants in human diets. The useful components from honeybee-collected pollen can be fully digested using the food-grade enzymes such as bromelain, cabbage aminopeptidase and proline iminopeptidase, and chick-pea aminopeptidase, although it is not easy to digest honeybee-collected pollen with a hard cell wall. In this process, consumer demand of honeybee-collected pollen for natural foods with medicinal effects such as antioxidative activity is increasing. 

In conclusion, pollen extracts represent a concentrated nature-derived mixture of different active polyphenol compounds which, according to practical applications as a bioactive diet component, are usually applied and consumed in higher amounts than the pure synthetic antioxidant food additives.

## Figures and Tables

**Table 1:** The contents of protein and total phenolic components of enzymatic hydrolysates from honeybee-collected pollen.

Sample	Protein (*μ*g/mg sample)	Total phenols (*μ*g/mg sample)	PI-value (%)
Bee-pollen	162.7 ± 0.2	15.3 ± 0.3	28 ± 2
^1^BH	227.1 ± 0.3	21.5 ± 0.6	40 ± 1
^2^APH_1_	238.8 ± 0.5	25.6 ± 0.7	44 ± 2
^3^APH_2_	230.5 ± 0.6	24.1 ± 0.9	42 ± 2
^4^APH_3_	242.8 ± 0.5	27.2 ± 0.6	46 ± 1

^1^BH bromelain hydrolysate

^2^APH_1_ cabbage aminopeptidase and proline iminopeptidase hydrolysate

^3^APH_2_ chick-pea aminopeptidase hydrolysate

^4^APH_3_ cabbage and chick-pea aminopeptidases hydrolysate

**Table 2:** The amino acids composition of honeybee-collected pollen and bee-pollen hydrolysate obtained by the combination of aminopeptidases from cabbage and chick-pea (*).

Amino acids	C (%)	C* (%)
Asp	7.5	6.6
Hyp	1.5	1.5
Glu	8.6	7.8
Ser	5.8	5.9
Gly	9.8	10.5
His + Thr	5.1	5.0
Ala	8.0	7.9
Arg	4.6	1.0
Pro	17.1	22.2
Tyr	1.7	1.4
Val	6.7	6.7
Met	2.2	2.1
Ile	5.3	5.1
Leu	7.9	7.5
Lys	3.4	2.6
Phe	8.1	5.5
